# Pathological Delta Oscillations in Hallucinogen Persisting Perception Disorder: A Case Report

**DOI:** 10.3389/fpsyt.2022.867314

**Published:** 2022-03-24

**Authors:** David Haslacher, Nikolina Novkovic, Maria Buthut, Andreas Heinz, Surjo R. Soekadar

**Affiliations:** Department of Psychiatry and Neurosciences, Clinical Neurotechnology Lab, Neuroscience Research Center, Charité – Universitätsmedizin Berlin, Berlin, Germany

**Keywords:** hallucinogen persisting perception disorder (HPPD), transcranial direct current stimulation (tDCS), delta oscillations, excitation/inhibition, EEG, non-invasive brain stimulation (NIBS)

## Abstract

**Background:**

Hallucinogen persisting perception disorder (HPPD) is characterized by spontaneous recurrence of visual hallucinations or disturbances after previous consumption of hallucinogens, such as lysergic acid diethylamide (LSD). The underlying physiological mechanisms are unknown and there is no standardized treatment strategy available.

**Case Presentation:**

A 33-year-old male patient presented with persistent visual distortions (halos around objects, intensified colors, positive after images, and trails following moving objects) that developed after repeated use of hallucinogenic drugs at the age of 18. Symptoms developed gradually and worsened several months later, resulting in various pharmacological and psychosocial treatment attempts that remained unsuccessful, however. At presentation, 32-channel electroencephalography (EEG) showed increased delta activity over the occipital brain regions, reminiscent of occipital intermittent rhythmic delta activity (OIRDA) usually seen in children. Two sessions of cathodal (inhibitory) transcranial direct current stimulation (tDCS) over 30 min attenuated visual hallucinations and occipital delta activity by approximately 60%. The response persisted for over four weeks.

**Conclusion:**

Pathological delta activity over occipital brain regions may play an important role in the development and perpetuation of HPPD and can be attenuated by non-invasive brain stimulation.

## Background

The consumption of hallucinogens such as lysergic acid diethylamide (LSD) or methylenedioxymethamphetamine (MDMA) can result in long-lasting and possibly permanent occurrence of perceptual, mainly visual, and disturbances. Prevalence of such hallucinogen-persisting perception disorder (HPPD) may further increase due to the growing diffusion of novel psychoactive substances able to cause the onset of the disorder (1, 2). HPPD was first described by Sandison et al. in the 1950s when the therapeutic value of LSD in mental illness was more systematically investigated (3). Commonly, two types of HPPD were described, an intermittent form characterized by spontaneous and brief recurrence of perceptual distortions or images (Type 1), and a permanent form where patients experience ongoing changes that can fluctuate in their intensity (Type 2) (4, 5). The disorder was later confirmed as a nosological entity, first as post-hallucinogenic perception disorder in the DSM-III-R and then later as HPPD in the DSM-IV-TR and DSM-5. According to DSM-5, the following criteria must be met to diagnose HPPD: (1) reexperiencing one or more of the perceptual symptoms that were experienced while intoxicated with the hallucinogens following cessation of hallucinogen use, (2) clinically significant distress due to (1), and (3) symptoms are not better accounted for by another disorder (e.g., delirium, dementia, schizophrenia) or general medical conditions (brain lesions and infections), and hypnopompic hallucinations occurring during state transition between sleep and wakefulness can be excluded.

While adverse reactions to hallucinogens are rare (6) and prevalence of HPPD relatively low (7), it can lead to severe suffering with episodes of major depression and suicidality (8). While there are some reports of successful pharmacological treatment with neuroleptics (9), anticonvulsants (10), benzodiazepines (11), or clonidine (12), there is no generally accepted treatment strategy available, and in some cases, pharmacological treatment has also been reported to exacerbate symptoms of HPPD (13). Depending on the specific psychopathology and possible neurological or psychiatric comorbidities, a combination of medications may be needed (14).

Currently, the underlying pathophysiological causes of HPPD are still widely unknown. Currently, a multifactorial origin of HPPD-related symptoms is assumed rendering a unified pathophysiological model difficult (15). Neurophysiological assessments using electroencephalography (EEG) could previously associate occipital hypersynchrony in the delta frequency band in HPPD (16) with reduced coherence of the occipital region to more distant cortical regions. Based on this finding, it was speculated that the primary visual cortex (V1) decouples from higher cortical areas in HPPD facilitating visual hallucinations. Such decoupling was also found in the context of palinopsia, i.e., reoccurrence of visual images after the stimulus is no longer present, e.g., due to a metastatic lesion of the occipitotemporal region (17). While occipital delta oscillations during wakefulness can be also seen in children, e.g., in the form of occipital intermittent rhythmic delta activity (OIRDA), their occurrence in the adulthood is typically appreciated as interictal phenomenon or described in the context of hallucination-prone dementias, such as Lewis-Body disease (18). It is unclear, however, whether occipital delta activity plays a causal role for visual distortions and hallucinations reported in HPPD.

## Case Presentation

A 32-year-old man presented with complaints of visual hallucinations that he characterized as “golden glitter” and “glowing translucent patches” in his total visual field. He also complained about seeing trails that follow moving objects. Particularly, loud colors, such as neon-colored objects or clothes, would intimidate him due to their intensity and could also trigger more severe episodes of visual distortions over several hours.

He reported that symptoms started gradually in the months after he repeatedly consumed hallucinogenic drugs at the age of 18. Due to the symptoms, he withdrew from social contacts and became increasingly insecure. At the age of 19, he sought professional help. Cranial magnetic resonance imaging (cMRI) remained unremarkable (except for a left cerebellar arachnoid cyst). A clinical EEG was interpreted as normal. To attenuate symptoms of depression with difficulties to sleep, he received citalopram and quetiapine that improved these symptoms, but not the visual hallucinations. About 8 years later, he developed an acute psychiatric crisis with anxiety and irritability as well as increased perceptual disturbances. During this time, he did not feel safe in his apartment anymore and had increasing suicidal ideations resulting in admission to a psychiatric hospital. At admission, he also mentioned distortions of sound for the time of one day that were classified as akoasms. The reported symptoms were interpreted as signs of an acute (schizophreniform) psychotic episode. During inpatient treatment, the daily dose of quetiapine was increased, which led to an improvement of symptoms. The leading tentative diagnoses over the coming years were a suspected schizophrenic psychosis that alternated with episodes of depression.

**FIGURE 1:**
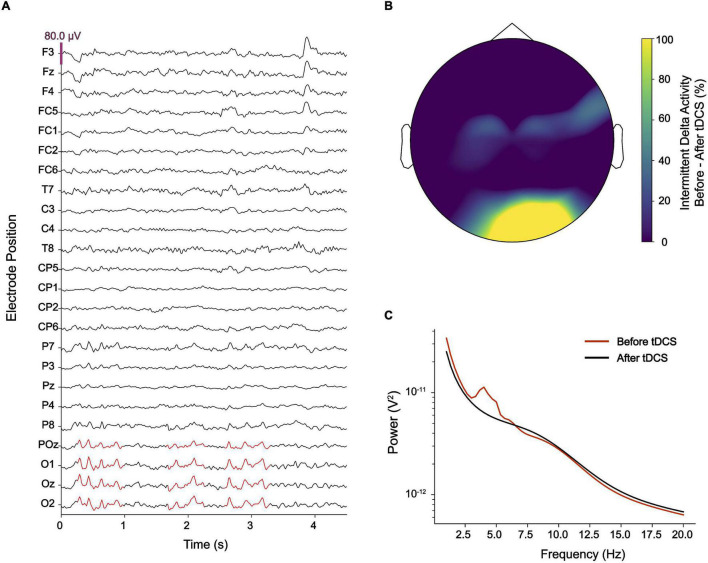
**(A)** Electroencephalography (EEG) showed intermittent pathological delta activity (marked in red) over occipital electrodes. **(B)** After delivering cathodal transcranial direct current stimulation (tDCS) over occipital brain regions, pathological delta activity became substantially reduced. **(C)** While an EEG power-spectrum averaged for occipital electrodes (O1, Oz, O2, and POz) showed a clear peak at ∼4 Hz, this peak disappeared after cathodal tDCS.

**FIGURE 2:**
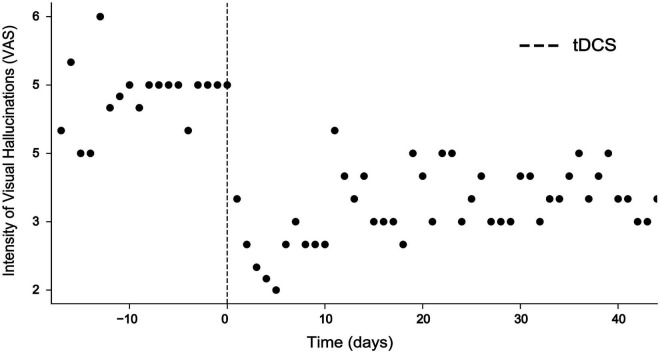
Intensity of visual hallucinations rated on a visual analog scale (VAS) ranging from 1 = minimal to 10 = extreme before and after intervention. While VAS scores ranged at around 5 before intervention, tDCS resulted in a highly significant decrease of VAS scores for 10 days (Mann–Whitney U test, *p* < 0.001***) and remained reduced for more than 40 days.

During this time, he became treated with various neuroleptics, including clozapine, haloperidol, amisulprid, ziprasidone, risperidone, quetiapine, aripiprazole, perazine, and promethazine, but did not experience any lasting improvement of visual hallucinations. Due to side-effects, including akathisia and other extrapyramidal motor symptoms (EPMS), neuroleptics were discontinued. Out of his despair, he contacted numerous physicians and therapists, including the Center for Neuromodulation at the Department of Psychiatry and Psychotherapy of the Charité – Universitätsmedizin Berlin where he was diagnosed with HPPD at the age of 32 based on his self-report and course of disease. A 32-channel EEG was performed and showed diffuse slowing of brain oscillations across broad areas of cortex with high power in the delta range over occipital electrodes (Figure 1A). Resting state EEG did not show a clear alpha-peak with eyes open and no alpha blockage during eyes closed. Delta activity did not exhibit any notable lateralization. The patient was asked to rate occurrence and intensity of visual hallucinations or distortions using a visual analog scale (VAS, ranging from 1 = minimal to 10 = extreme) three times a day daily (Figure 2).

Based on the large body of literature showing reduced cortical excitability after cathodal transcranial direct current stimulation (tDCS) (19, 20), we delivered tDCS at 2 mA to the occipital lobe using large rubber electrodes (5 cm × 7 cm). The electrodes were placed perpendicularly to the midline, such that the cathode was centered to electrode position Oz and the anode was centered to position Fpz according to the international 10–20 system. Two 30-min sessions of tDCS each were performed, separated by an intermission of 30 min. EEG was obtained immediately before the first and after the second session of tDCS.

During the session, the patient reported that his visual hallucinations gradually improved. After the second session, the patient reported an improvement of visual hallucinations or distortions by approximately 60% which sustained for 10 days (Figure 2). A Mann–Whitney *U* test comparing baseline VAS values provided for the 10 days before intervention with VAS values provided for the 10 days after intervention was highly significant (*p* < 0.001). Evaluation of the EEG recorded after the second session showed that abnormal delta activity over the occipital region disappeared (Figures 1B,C). Over the coming 4 weeks attenuation of delta activity remained reduced by approximately 40% relative to the pre-tDCS level (Figure 2). During this time, also symptoms of depression improved, and the patient reported alleviation of anxiety and agitation. Although visual distortions did not completely disappear and their intensity remained at a level of 3–4 on a VAS, the patient underlined that he felt great relief because tDCS provided him a mechanistic tool to control his symptoms.

## Discussion and Conclusion

In the presented case diagnosed with HPPD, we found that pathological delta activity was causally linked to visual hallucinations. While occipital delta hypersynchrony was described earlier in HPPD (16), this is the first case report in which clinical symptoms were successfully treated with non-invasive brain stimulation to attenuate such pathological delta activity.

It has been proposed that symptoms of HPPD are caused by damage to inhibitory interneurons expressing 5-HT_2A_ serotonin receptors to which most hallucinogens bind. This loss of cortical inhibition (21, 22) may manifest in aberrant occipital delta oscillations (23–25) associated with visual hallucinations. Our results support such model and suggest that disinhibition can be restored with cathodal (inhibitory) tDCS. Future studies should investigate whether the reported findings can be generalized to larger cohorts diagnosed with HPPD.

While two sessions of cathodal tDCS had an immediate effect on clinical symptoms and pathological delta activity, the exact mechanisms by which tDCS affected pathological delta activity are not entirely clear.

The most plausible and accepted primary effect of tDCS relates to modulation of resting membrane potentials (RMP), with anodal stimulation resulting in decreased RMP and cathodal stimulation in increased RMP reducing likelihood of action potentials (26, 27). It was shown, however, that this primary effect can result in a multitude of other effects, e.g., on neurogenesis and microglia activation (28), that may explain the outlasting impact of tDCS on brain excitability and clinical symptoms. In our case, stimulation effects sustained for more than 40 days with a maximum within the first 12 days. It is unclear whether more tDCS sessions would have further improved clinical symptoms, a question we could not investigate due to the distance to the patient’s place of residence.

After an initial clinical EEG was interpreted as normal and was not repeated later over the course of treatment, our case underlines the necessity for EEG re-assessments in the management of neuropsychiatric disorders. Since the patient developed a psychiatric crisis 8 years after the onset of symptoms that was interpreted as acute schizophreniform psychosis, the following treatment regime focused on the use of various neuroleptic agents. Since comorbidity may often occur in severe forms of HPPD, including anxiety and depression with psychotic symptoms that may lead to the first admission to a psychiatric hospital, proper assessment of patient history and drug use is essential to disentangle different nosological entities. In this context, it is also possible that the psychotic episode and occurrence of HPPD had a common background.

In the presented case, cathodal tDCS did not completely terminate visual distortions, but resulted in considerable symptom relief opening a window for psychosocial interventions, such as cognitive behavioral therapy that was previously ineffective because of symptom severity. The experience of symptom control using non-invasive neuromodulation could end the vicious circle of stress and anxiety also described in other neuropsychiatric cases, e.g., tDCS treatment of multimodal hallucinations (29).

While the presented case suggests a causal role of occipital delta oscillations for the occurrence of visual distortions or hallucinations in HPPD, millisecond-to-millisecond precise targeting of these oscillations using closed-loop transcranial alternating current stimulation (cl-tACS), e.g., to purposefully suppress such activity, could further corroborate this causal relationship. While establishing such paradigm is very challenging due to stimulation artifacts, recent advances in stimulation artifact suppression may render such approach feasible (30).

## Data Availability Statement

The raw data supporting the conclusions of this article will be made available by the authors, without undue reservation.

## Ethics Statement

Ethical review and approval was not required for the study on human participants in accordance with the local legislation and institutional requirements. The patient provided his written informed consent to participate in this study.

## Author Contributions

DH performed tDCS treatment, data analysis, and wrote the manuscript. NN performed neuropsychological evaluation, data analysis, and wrote the manuscript. MB and AH evaluated the medical history and revised the manuscript. SRS performed neuropsychological evaluation and wrote the manuscript. All authors contributed to the article and approved the submitted version.

## Conflict of Interest

The authors declare that the research was conducted in the absence of any commercial or financial relationships that could be construed as a potential conflict of interest.

## Publisher’s Note

All claims expressed in this article are solely those of the authors and do not necessarily represent those of their affiliated organizations, or those of the publisher, the editors and the reviewers. Any product that may be evaluated in this article, or claim that may be made by its manufacturer, is not guaranteed or endorsed by the publisher.

## Abbreviations

tDCS: transcranial direct current stimulation
EEG: electroencephalogram
HPPD: hallucinogen persistent perception disorder
MDMA: methylenedioxymethamphetamine
LSD: lysergic acid diethylamide
OIRDA: occipital intermittent rhythmic delta activity.
